# Fibrin‐targeting delivery: a novel platform for cardiac regenerative medicine

**DOI:** 10.1111/jcmm.12912

**Published:** 2016-07-29

**Authors:** Zheyong Huang, Yanan Song, Zhiqing Pang, Minghui Li, Yerkintay Guliya, Yunli Shen, Juying Qian, Junbo Ge

**Affiliations:** ^1^ Shanghai Institute of Cardiovascular DiseasesZhongshan HospitalFudan UniversityShanghaiChina; ^2^ School of PharmacyFudan UniversityKey Laboratory of Smart Drug DeliveryMinistry of EducationShanghaiChina; ^3^ Department of CardiologyShanghai East HospitalTongji UniversityShanghaiChina

**Keywords:** fibrin, CREKA, cell therapy, targeting therapy, myocardial infarction

## Introduction and background

Myocardial infarction (MI) and subsequent heart failure secondary to the massive lost of cardiomyocyte is a major cause of morbidity and mortality world‐wide. Given the limited endogenous potential for renewal of cardiomyocytes in adults, cardiac cell‐based therapies generating new cardiomyocytes and vessels have emerged as a promising treatment to reverse functional deterioration and prevent the progression to CHF. Although different type of stem cells, including mesenchymal stem cells (MSCs), CD34^+^ cells and cardiac‐derived stem cells, have been clinically tried, poor cell homing, retention and engraftment remain major obstacles to achieve a significant functional benefit 1, 2, 3. It has long been a hot issue to find an effective homing target in cardiac cellular therapy.

To direct transplanted stem cells to the injured heart, we and other groups sought to cellular magnetic targeting, which did enhance the therapeutic effects of stem cells through increasing cell retention in ischaemia/reperfusion rat models 4, 5, 6, 7. However, the external magnetic force can only promote a transient and imprecise stay in the heart, with a hidden risk of unfavourable vascular embolization and an inhomogeneous distribution of the donor cells 8, 9. Therefore, the precise cell homing to the injured myocardium depends on the biological homing mechanisms.

The first prerequisite for biologically targeting therapy is to find a good target element, which should exclusively express in the injured region or at least distribute in a concentration‐gradient pattern. A rapid and dynamic change occurs in the local myocardial microenvironment following MI. Cytokine network system, myocardial parenchymal cell components and extracellular matrix may all serve as a possible candidate target after the injury of myocardium. Much efforts have been made in identifying chemokine and its receptors (CXCR4/SDF‐1 axis, *etc*.) in the last decades 10. However, the cytokine‐based method has been far away from being able to effectively regulate stem cells migrating to target tissue due to the extremely complicity of the cytokine interaction and the homing molecular mechanisms 11, 12. Recently, components released from injured cardiomyocyte (such as myosin light chain, actin and myosin associated proteins) have been explored as novel homing targets 13, 14, 15, 16. However, cardiomyocyte components may also release into the bloodstream, weakening the specificity of tissue distribution. Then, how about the performance of the extracellular matrix proteins? Actually, extracellular matrix proteins, as a possible cell homing target, has long been neglected in the field of cardiovascular disease.

Fibrin is a fibrous protein involved in the tissue healing, blood clotting and tumour invasion. Tissue injury and healing (such as MI) witness the dynamic changes in the composition of the extracellular matrix. Increased vascular permeability results in extensive extravasation of plasma proteins that form a fibrin‐based provisional matrix, providing the scaffold for the infiltration of granulation tissue cells 17. With the organization of the injury, the fibrin network is lysed and replaced by fibronectin, hyaluronan, matricellular proteins and collagen. In tumour tissues, tumour cells continue to secrete vascular permeability factor (*i.e*. VEGF‐A and VEGF), the fibrinogen was secreted into the stroma of malignant lesions to form a large number of fibrin 18. Taking advantage of the fibrin deposition that is characteristic of tumours, Chung *et al*. constructed special chemotherapeutic agents with the fibrin‐binding peptide, which facilitate efficient delivery of chemotherapeutic agents to malignant gliomas while minimizing systemic toxicity and side effects 19. These findings suggest that the fibrin‐targeting therapy may be a generalizable platform technology for tissue injury, because the fibrin‐based provisional matrix is a basic pathologic process in tissue healing.

Based on the spatiotemporal pattern of the expression of fibrin after myocardial ischaemia/reperfusion injury, fibrin satisfies the basic requirement as a homing target molecule in regenerative medicine. From the space angles, fibrin only deposits in the infarcted area, while rarely fibrin exists in the healthy myocardium 20, 21. So the fibrin deposition region is exactly in accordant with the target region of regenerative therapy. From the perspective of timing, fibrin forms immediately after cardiac myocyte necrosis in the setting of myocardial injury, and fibrin deposition was transient and followed by formation of a mature collagen‐based scar after 7–14 days of reperfusion in a mouse model of reperfused infarction 20. The duration of the fibrin is coincident with the optimal timing of cell transplantation, which was recently suggested as the first week after MI 22, 23. In addition, fibrin has pleiotropic effects on issue repair, including mediating the migration of inflammatory cells and the proliferation of stem cells 24, 25. What is more, fibrin‐based bioscaffolds have been extensively applied in stem cell therapeutics and the cardiac muscle tissue engineering 26, 27. Therefore, the spatiotemporal characteristics of fibrin expression after MI, including specifically appearance in the infarcted area spatially and in early repair phase temporarily, along with its pleiotropic effects, render fibrin a perfect target for the delivery of stem cells.

Another prerequisite for biologically targeting therapy is to endow the therapeutic cells with the specific and efficient binding capacity with the target component. It can be fulfilled by ligand modification of cell membrane. Although antibody has been traditionally used as the fibrin‐targeting ligand in detecting fibrin component within thrombus 28. It is believed that peptide modification is superior to the antibody technology, due to its relatively simple structure, convenient synthesis, high purity and high capacity linking ability with cells 29.

Clot‐binding peptide cysteine–arginine–glutamic acid–lysine–alanine (CREKA) is a tumour‐homing pentapeptide obtained by *in vivo* phage display in MMTV‐PyMT Transgenic breast cancer mice. CREKA has been shown to target various cancers by binding to fibrin and fibrin‐associated clotted plasma proteins within the tumour vasculature 30, 31, 32. Furthermore, fluorescently labelled micelles functionalized with the CREKA pentapeptide were reported to home to sites of fibrin found on thrombotic, atherosclerotic plaques 33. Recently, we also conjugated CREKA with fluorescence‐labelled superparamagnetic iron oxide nanoparticles, yielding a novel thrombus‐targeting nanoagent for multimodal imaging of microthrombus, which can be detected by both magnetic resonance and optical imaging modalities 34. The increasing evidence in the molecular targeted imaging/therapy proved out that CREKA is reasonably expected to serve as a reliable ligand peptide for fibrin in myocardial injury.

## Hypothesis

**Figure 1:**
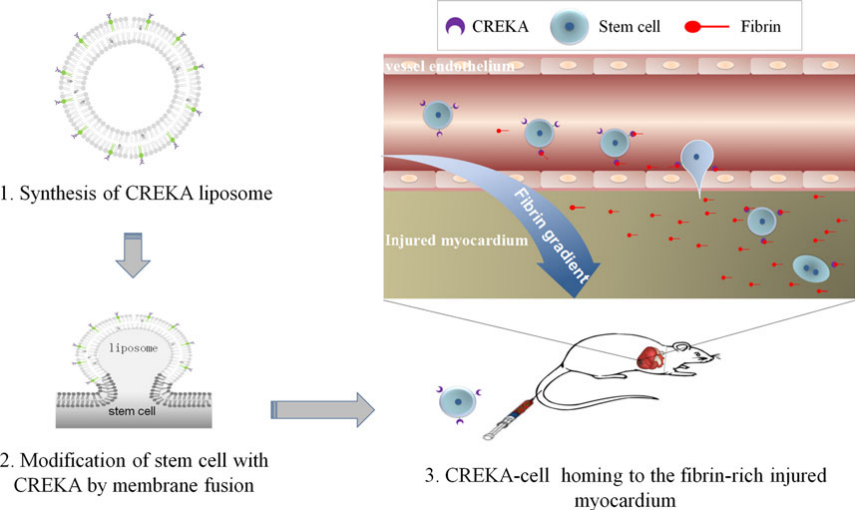
Cartoon to illustrate how the ‘stem cell‐CREKA‐fibrin’ targeting system recruit the exogenous stem cells to the injured and fibrin‐rich heart.

Based on available studies, it is logical to hypothesize that fibrin should be a potential and new target in the stem cell therapy for the MI, and the ‘stem cells‐CREKA‐fibrin’ targeting system may recruit the exogenous stem cells to the injured and fibrin‐rich heart. The hypothesis could be verified in animal study. First, the phosphorylated CREKA is connected to liposome membrane, and then stem cells (such as bone marrow MSCs, *etc*.) were coated with CREKA peptides by using the liposome membrane fusion technology and the fluidity of the lipid bilayer. The affinity of fibrin to the CREKA‐modified stem cells and the stability of the connection between CREKA and stem cells are evaluated. Second, the CREKA modified stem cells are to be injected into the left ventricle or the tail vein of the myocardial ischaemia‐reperfusion model of rats, and the transplanted cells will direct to the fibrin deposit in the injured region to improve cardiac performance (Fig. 1). Notably, the addition of surface modifications by membrane fusion technology, while useful, has potential to impair functions of membrane proteins or even trigger signalling events when the surfaces are densely modified thereby potentially altering receptor binding efficiency. Therefore, the potential cytotoxicity of CREKA coating should carefully be evaluated.

## Implication

Fibrin offers a brand‐new target for transplanted cells homing to the injured myocardium. Different from external magnetic targeting, fibrin targeting represents a biological and precise homing to the injured region because of the spatial‐specific distribution of fibrin following myocardial injury. To the best of our knowledge, it is the first time to propose a component of the extracellular matrix as cell target in the field of cardiovascular diseases, providing new perspectives for the possibility of other extracellular matrix production (such as fibronectin) as biologically therapeutic target. To effectively overcome the shortcoming of low homing in cell transplantation for MI and heart failure, more benefits may be expected from the combination of different homing strategy (such as extracellular matrix targeting, intrinsic cytokine targeting and external magnetic guidance).

More importantly, the fibrin‐targeting strategy is a generalizable platform technology for regenerative medicine. On the one hand, any therapeutic agents (not only exogenous stem cells but also drug *etc*.) can be directed to damaged and fibrin‐rich heart, as long as it can be combined with CREKA peptide. For example, targeting reparative factors and microRNA to the injured heart promotes the efficacy of cardiomyocyte proliferation and cardiac repair and transcription factors promotes reprogramming of cardiac fibroblasts towards cardiomyocytes. On the other hand, microvascular hyperpermeability and the introduction of a plasma‐derived, fibrin‐based provisional matrix is a basic pathologic characteristic in virtually almost all tissue injury 17, raising the possibility that fibrin‐targeting technique may also be indicated in other tissue injury with the presence of rich fibrin at the early stage, in addition to MI.

In conclusion, the spatiotemporal distribution pattern makes fibrin a potential and new target in the stem cell therapy for the MI, and the ‘stem cells‐CREKA‐fibrin’ targeting system may localize the exogenous stem cells to the injured and fibrin‐rich heart, subsequently enhance the efficacy of stem cell therapy.

## Conflict of interest

The authors indicate no potential conflicts of interest.
